# Supplementary material for: Does quality of life return to pre-treatment levels five years after curative intent surgery for colorectal cancer? Evidence from the ColoREctal Wellbeing (CREW) study
Source: PLoS One. 2020 Apr 9;15(4):e0231332. doi: 10.1371/journal.pone.0231332 (PMC7145191; doi:10.1371/journal.pone.0231332)
Supplement: S1 Table — (DOCX) [file pone.0231332.s001.docx]

**S1 Table**

| Time-point | List of Threatening Experiences | SCNS-SF34 | EQ5D | PWI | CES-D | QLACS Part I | QLACS Part II | EORTC QLQ-C30 | EORTC QLQ-CR29 | MOS-SSS | STAI | SEMCD | CS-SES | PANAS scale | COPE | IPQ-R | SCIN |
| --- | --- | --- | --- | --- | --- | --- | --- | --- | --- | --- | --- | --- | --- | --- | --- | --- | --- |
| Pre-surgery | X | X | √ | √ | √ | √ | X | X | X | √ | √ | √ | X | √ | √ | X | X |
| 3m | √ | X | √ | √ | √ | √ | X | √ | √ | √ | √ | √ | X | √ | √ | X | X |
| 9m | √ | X | X | √ | √ | √ | √ | √ | √ | √ | √ | √ | X | √ | √ | X | X |
| 15m | √ | √ | √ | √ | √ | √ | √ | √ | √ | √ | √ | √ | √ | √ | √ | √ | X |
| Brief 15m | X | X | √ | √ | X | √ | √ | X | X | X | X | √ | X | X | X | X | X |
| 24m | √ | √ | √ | √ | √ | √ | √ | √ | √ | √ | √ | √ | √ | √ | √ | X | X |
| Brief 24m | X | X | √ | √ | X | √ | √ | X | X | X | X | √ | X | X | X | X | X |
| 36m | √ | X | √ | √ | √ | √ | √ | √ | √ | √ | X | √ | √ | X | X | X | √ |
| Brief 36m | √ | X | √ | √ | X | √ | √ | √ | X | X | X | √ | √ | X | X | X | X |
| 48m | √ | X | √ | √ | √ | √ | √ | √ | √ | √ | X | √ | √ | X | X | X | √ |
| Brief 48m | √ | X | √ | √ | X | √ | √ | √ | X | X | X | √ | √ | X | X | X | X |
| 60m | √ | X | √ | √ | √ | √ | √ | √ | √ | √ | √ | √ | √ | X | √ | X | √ |
| Brief 60m | √ | X | √ | √ | X | √ | √ | √ | X | X | X | √ | √ | X | X | X | X |
