# Supplementary material for: Does quality of life return to pre-treatment levels five years after curative intent surgery for colorectal cancer? Evidence from the ColoREctal Wellbeing (CREW) study
Source: PLoS One. 2020 Apr 9;15(4):e0231332. doi: 10.1371/journal.pone.0231332 (PMC7145191; doi:10.1371/journal.pone.0231332)
Supplement: S3 Table — (DOCX) [file pone.0231332.s003.docx]

**S3 Tables**

**Colon**

| **Thematic Block** | **Measure** | **Time point** | |
| --- | --- | --- | --- |
|  |  | **Baseline** | **24m** |
| **Pre-existing factors (Socio-demographics)** | Age | + | + |
|  | Gender | + | baseline |
|  | Employment Status (employed vs. not) | + | + |
|  | Deprivation Index | + | baseline |
| **Clinical factors** | Duke's stage | + | baseline |
|  | Surgery type (open vs laproscopic) | + | baseline |
|  | Stoma | + | - |
|  | Adjuvant chemotherapy | + | + |
|  | Comorbidities | 3m | + |
|  | Body Mass Index (BMI) | + | baseline |
|  | Domestic status | + | + |
| **Environmental factors** | List of Threatening Experiences | - | + |
|  | Medical Outcome Study Social Support Scale (MOS-SSS) | + | + |
|  | Self-Efficacy for Managing Chronic Disease 6-Item Scale (SEMCD) | + | + |
| **Personal factors** | Cancer Survivor Self-Efficacy Scale (CS-SES) | - | + |
|  | Positive and Negative Affect Schedule Short Form (PANAS-SF) | + | + |
|  | Centre for epidemiological studies depression (CES-D) | + | + |
|  | European Organization for Research and Treatment of Cancer (EORTC) QLQ-C30 and QLQ-CR29 scales: physical functioning, cognitive functioning, emotional functioning, pain, dyspnoea, diarrhoea, fatigue, urinary frequency | - | + |
|  |  |  |  |
| **Psychosocial outcomes** | State-Trait Anxiety Inventory - State (STAI-S) | + | + |
|  | Personal Wellbeing Index - Adult (PWI-A) | + | + |
|  | EQ-5D full health status | + | + |
|  | Quality of Life in Adult Cancer Survivors - Generic Summary Score (QLACS-GSS) | + | + |
|  | Quality of Life in Adult Cancer Survivors - Cancer-Specific Summary Score (QLACS-CSS) | - | + |
|  | QLACS Benefit of Cancer | - | + |

**Rectal**

| **Thematic Block** | **Measure** | **Time point** | |
| --- | --- | --- | --- |
|  |  | **Baseline** | **24m** |
| **Pre-existing factors (Socio-demographics)** | Age | + | + |
|  | Gender | + | baseline |
|  | Employment Status (employed vs. not) | + | + |
|  | Deprivation Index | + | baseline |
| **Clinical factors** | Duke's stage | + | baseline |
|  | Surgery type (open vs laproscopic) | + | baseline |
|  | Stoma | + | + |
|  | Neoadjuvant chemotherapy | + | + |
|  | Neoadjuvant radiotherapy | + | + |
|  | Adjuvant chemotherapy | + | + |
|  |  |  |  |
|  | Comorbidities | 3m | + |
|  | Body Mass Index (BMI) | + | baseline |
|  |  |  |  |
| **Environmental factors** | Domestic status | + | + |
|  | List of Threatening Experiences | - | + |
|  | Medical Outcome Study Social Support Scale (MOS-SSS) | + | + |
| **Personal factors** | Self-Efficacy for Managing Chronic Disease 6-Item Scale (SEMCD) | + | + |
|  | Cancer Survivor Self-Efficacy Scale (CS-SES) | - | + |
|  | Positive and Negative Affect Schedule Short Form (PANAS-SF) | + | + |
|  | Centre for epidemiological studies depression (CES-D) | + | + |
| **Psychosocial outcomes** | European Organization for Research and Treatment of Cancer (EORTC) QLQ-C30 and QLQ-CR29: physical functioning, cognitive functioning, emotional functioning, social functioning, pain, dyspnoea, diarrhoea, fatigue, insomnia, nausea/vomiting, urinary frequency, stool frequency, flatulence, anxiety | - | + |
|  | State-Trait Anxiety Inventory - State (STAI-S) | + | + |
|  | Personal Wellbeing Index - Adult (PWI-A) | + | + |
|  | EQ-5D full health status | + | + |
|  | Quality of Life in Adult Cancer Survivors - Generic Summary Score (QLACS-GSS) | + | + |
|  | Quality of Life in Adult Cancer Survivors - Cancer-Specific Summary Score (QLACS-CSS) | - | + |
|  | QLACS Benefit of Cancer | - | + |

- variable not included in model

+ variable from contemporary time point included

baseline/3m: variable from specified time point included in the model
