# Supplementary material for: Does quality of life return to pre-treatment levels five years after curative intent surgery for colorectal cancer? Evidence from the ColoREctal Wellbeing (CREW) study
Source: PLoS One. 2020 Apr 9;15(4):e0231332. doi: 10.1371/journal.pone.0231332 (PMC7145191; doi:10.1371/journal.pone.0231332)
Supplement: S4 Table — (DOCX) [file pone.0231332.s004.docx]

**S4 Table**

**Multivariable logistic regression models with odds ratios of worsened QOL (ref: improved/same QOL) with statistically significant factors taken separately from two time points for people with colon cancer**

| **Covariates** | **Model 1: Baseline** | **Model 2: 24 m** |
| --- | --- | --- |
|  | **OR (95% CI)** | **OR (95% CI)** |
| Age groups (ref: 60 or younger) |  |  |
| 61-70 |  | 2.92 (1.32; 6.43) |
| 71-80 |  | 3.27 (1.42; 7.54) |
| 81+ |  | 5.71 (2.01; 16.26) |
|  |  |  |
| BMI (ref: BMI<30) |  |  |
| BMI>30 | 2.31 (1.44; 3.72) | 2.18 (1.22; 3.90) |
| Missing | 1.03 (0.65; 1.61) | 1.21 (0.70; 2.10) |
|  |  |  |
| Stoma (ref: no stoma) | 2.25 (1.30; 3.91) |  |
|  |  |  |
| PANAS positive score |  | 0.88 (0.82; 0.95) |
|  |  |  |
| QLACS Cancer-specific score |  | 1.03 (1.01; 1.05) |
|  |  |  |
| Depression CES-D>=20 (ref: <20) | 2.07 (1.18; 3.64) |  |
|  |  |  |
| Self-efficacy (ref: low confidence) |  |  |
| - moderate confidence | 0.39 (0.18; 0.84) | 0.33 (0.12; 0.91) |
| - confident | 0.33 (0.16; 0.66) | 0.55 (0.22; 1.40) |
| - very confident | 0.19 (0.08; 0.42) | 0.33 (0.11; 0.98) |
|  |  |  |
| Scoring above threshold for clinical importance on QLQ-C30 fatigue scale (ref: not) |  | 2.16 (1.02; 4.55) |
| Scoring above threshold for clinical importance on QLQ-CR29 urinary frequency scale (ref: not) |  | 1.92 (1.17; 3.17) |
| N observations (N individuals) | 1216 (373) | 583 (228) |

**Multivariable logistic regression models with odds ratios of worsened QOL (ref: improved/same QOL) with statistically significant factors taken separately from two time points for people with rectal cancer**

| **Covariates** | **Model 1: Baseline** | **Model 2: 24 m** |
| --- | --- | --- |
|  | **OR (95% CI)** | **OR (95% CI)** |
| Age groups (ref: 60 or younger) |  |  |
| 61-70 | 0.66 (0.37; 1.18) |  |
| 71-80 | 0.36 (0.19; 0.67) |  |
| 81+ | 1.01 (0.32; 3.14) |  |
|  |  |  |
| Deprivation index (ref: Least deprived - 1st quintile) |  |  |
| 2nd quintile | 1.35 (0.64; 2.84) |  |
| 3rd quintile | 2.27 (1.02; 5.05) |  |
| 4th quintile | 1.07 (0.50; 2.26) |  |
| Most deprived - 5th quintile | 2.79 (1.17; 6.64) |  |
|  |  |  |
| Neo-adjuvant therapy (ref: none) | 2.41 (1.48; 3.94) |  |
|  |  |  |
| Comorbidities (ref: none) |  |  |
| one | 2.01 (1.08; 3.72) |  |
| 2+ | 1.86 (0.96; 3.63) |  |
|  |  |  |
| PANAS negative score | 1.12 (1.03; 1.210 |  |
|  |  |  |
| Scoring above threshold for clinical importance on QLQ-C30 cognitive functioning scale (ref: not) |  | 3.91 (1.69; 9.05) |
|  |  |  |
| 1+ Life events (ref:none) |  | 0.42 (0.22; 0.81) |
| N observations (N individuals) | 682 (207) | 344 (142) |

*Note*: * p<.05; ** p<.01; *** p<.001; N/A = not available at this time-point; empty cells indicate statistical insignificance (p>=.05); the wave of participation was statistically significant in each model (not presented); the original QLACS-GSS score was accounted for to control for the floor and ceiling effects, which was significant in each model (not presented).
